# A Comprehensive Description of the Anatomy and Histochemistry of *Psychotria capillacea* (Müll. Arg.) Standl. and an Investigation into Its Anti-Inflammatory Effects in Mice and Role in Scopolamine-Induced Memory Impairment

**DOI:** 10.3390/ph17050564

**Published:** 2024-04-28

**Authors:** Anelise Samara Nazari Formagio, Wagner Vilegas, Cândida Aparecida Leite Kassuya, Valter Paes De Almeida, Jane Manfron, Elisabete Castelon Konkiewitz, Edward Benjamin Ziff, Janaine Alberto Marangoni Faoro, Jessica Maurino Dos Santos, Ana Julia Cecatto, Maria Helena Sarragiotto, Rosilda Mara Mussury

**Affiliations:** 1 Faculty of Health Sciences, Federal University of Grande Dourados, Dourados 79825-070, MS, Brazil; aneliseformagio@ufgd.edu.br (A.S.N.F.); candidakassuya@ufgd.edu.br (C.A.L.K.); elisabetekonkiewitz@ufgd.edu.br (E.C.K.); janaine_dec4@hotmail.com (J.A.M.F.); jessicamaurinodossantos@gmail.com (J.M.D.S.); 2 Institute of Biosciences, São Paulo State University—UNESP, São Vicente 11330-900, SP, Brazil; wagner.vilegas@unesp.br; 3 Posgraduate Program in Pharmaceutical Sciences, State University of Ponta Grossa, Ponta Grossa 84010-330, PR, Brazil; valterp_almeida@hotmail.com (V.P.D.A.); jane@uepg.br (J.M.); 4 Department of Biochemistry and Molecular Pharmacology, NYU Grossman School of Medicine, New York University, New York City, NY 10012, USA; edward.ziff@icloud.com; 5 Department of Chemistry, State University of Maringá, Maringá 87020-900, PR, Brazil; anajulia.cecatto@gmail.com (A.J.C.); mhsarragiotto@gmail.com (M.H.S.); 6 Faculty of Biological and Environmental Sciences, Federal University of Grande Dourados—UFGD, Dourados 79825-070, MS, Brazil

**Keywords:** coffee, alkaloids, carrageenan, acetylcholinesterase, neuroprotective effect, scopolamine, microscopy

## Abstract

Species of the genus *Psychotria* are used in popular medicine for pain, inflammatory symptoms, and mental disorders. *Psychotria capillacea* (Müll. Arg.) Standl. (Rubiaceae) is commonly known as coffee and some scientific studies have demonstrated its therapeutic potential. The goal of this study was to investigate the anti-inflammatory and neuroprotective effects, and acetylcholinesterase (AChE) inhibitory activity of a methanolic extract obtained from leaves of *P. capillacea* (MEPC), as well as the micromorphology and histochemistry of the leaves and stems of this plant. In addition, the MEPC was analyzed by UHPLC-MS/MS and the alkaloidal fraction (AF) obtained from the MEPC was tested in a mouse model of inflammation. MEPC contained three indole alkaloids, one sesquiterpene (megastigmane-type) and two terpene lactones. MEPC (3, 30 and 100 mg/kg) and AF (3 and 30 mg/kg) were evaluated in inflammation models and significantly inhibited edema at 2 h and 4 h, mechanical hyperalgesia after 4 h and the response to cold 3 h and 4 h after carrageenan injection. Scopolamine significantly increased the escape latency, and reduced the swimming time and number of crossings in the target quadrant and distance, while MEPC (3, 30 and 100 mg/kg), due to its neuroprotective actions, reversed these effects. AChE activity was significantly decreased in the cerebral cortex (52 ± 3%) and hippocampus (60 ± 3%), after MEPC administration. Moreover, micromorphological and histochemical information was presented, to aid in species identification and quality control of *P. capillacea*. The results of this study demonstrated that *P. capillacea* is an anti-inflammatory and antihyperalgesic agent that can treat acute disease and enhance memory functions in mouse models.

## 1. Introduction

Many plant-derived natural products have been described as potent anti-inflammatory agents that may provide alternative strategies for the development of new drugs. *Psychotria capillacea* (Müll. Arg.) Standl. Is a shrub that belongs to the family Rubiaceae, subfamily Rubioideae, tribe Psychotrieae and subgenus Heteropsychotria [1]; it is popularly known as coffee and grows in the Brazilian states of Amazonas, Mato Grosso do Sul, and Parana, as well as in Paraguay and Argentina [2].

According to a preliminary screening by our research group, methanolic extracts obtained from the aerial parts (leaves and stems) of *P. capillacea* exhibit antioxidant activity according to the DPPH (IC_50_ = 30.05 μg/mL), linoleic acid peroxidation (33.40 μg/mL) and ABTS (87.34%) assays; these activities may be correlated with the high content of total phenolics (148.42 mg/g), flavonoids (91.58 mg/g), flavonols (185.54 mg/g) and the presence of p-coumaric acid according to high-performance liquid chromatography/photodiode array (HPLC/PDA) analyses [3]. In a second investigation, this extract showed a promising antiproliferative effect against ovarian OVCAR-3 (total growth inhibition of 2.33 µg/mL) and glioma U251 (16.66 µg/mL) cells and acetylcholinesterase (AChE) inhibition (21% in the hippocampus) in the brain structures of rats; the total alkaloids’ content was quantified to be 25.4 µg/g [4]. To our knowledge, these are the only studies described in the scientific literature.

Species of the genus *Psychotria* are used in popular medicine for pain- and inflammation-related symptoms [5,6] and mental disorders [7]. According to a literature review of pharmacological studies carried out with extracts from leaves of this genus, these exhibit several biological effects, including anti-inflammatory and analgesic effects [8,9] and activities that protect the central nervous system [9,10,11]. Preliminary tests revealed that alkaloids (87% of which are indole alkaloids) were the major compounds responsible for the effects, in addition to triterpenes (12%), flavonoids (6%), and constituents of other classes; i.e., indole alkaloids (strictosidine and 5α-carboxystrictosidine) and triterpenes (pomolic acid and spinosic acid) isolated from *P. nuda*, which showed promising inhibitory activity against nitric oxide (NO) production [12]; alkaloids isolated from *P. nemorosa* (nemorosinoside G, serotonin and bufotenine), which displayed anticholinesterase activity [10]; nemoroside and fargesine, which were able to significantly extend the time of metallothionein induction, a process that is associated with reduced neurodegeneration of aged brain tissue [13]; vincosamide isolated from *P. leiocarpa*, which showed anti-inflammatory activity and AChE inhibitory effects [11]; alkaloids, obtucarbamate A, and the triterpene asperulosidic acid isolated from *P. prainii*, which showed anti-inflammatory activity [14] and strictosidinic acid isolated from *P. Myriantha*, which inhibited monoamine oxidase activity in the rat hippocampus [15].

Dementia comprises a set of symptoms in elderly individuals including pronounced memory loss. Alzheimer’s disease (AD) accounts for 60–70% of dementia cases [16], and more than half of individuals with AD experience recurring pain [17,18]. Pain is known to affect cognitive dysfunction in humans and animals, including deficits in attention [19], executive function [20], and memory [21].

Thus, *Psychotria* species have several uses in popular medicine including for the treatment of pain and mental disorders, which prompted research on *P. capillacea.* The aim of this study was to investigate the anti-inflammatory (effects on pain), neuroprotective (ability to prevent emotional and spatial memory) effects and AChE inhibitory activity of the methanolic extract obtained from leaves of *P. capillacea* (MEPC), as well as the anatomy of the leaves and stems this plant. In addition, the MEPC was analyzed by UHPLC-MS/MS and alkaloidal fraction (AF) was tested in a mouse model of inflammation.

## 2. Results

### 2.1. Micromorphology and Histochemistry Analysis

**Figure 1:**
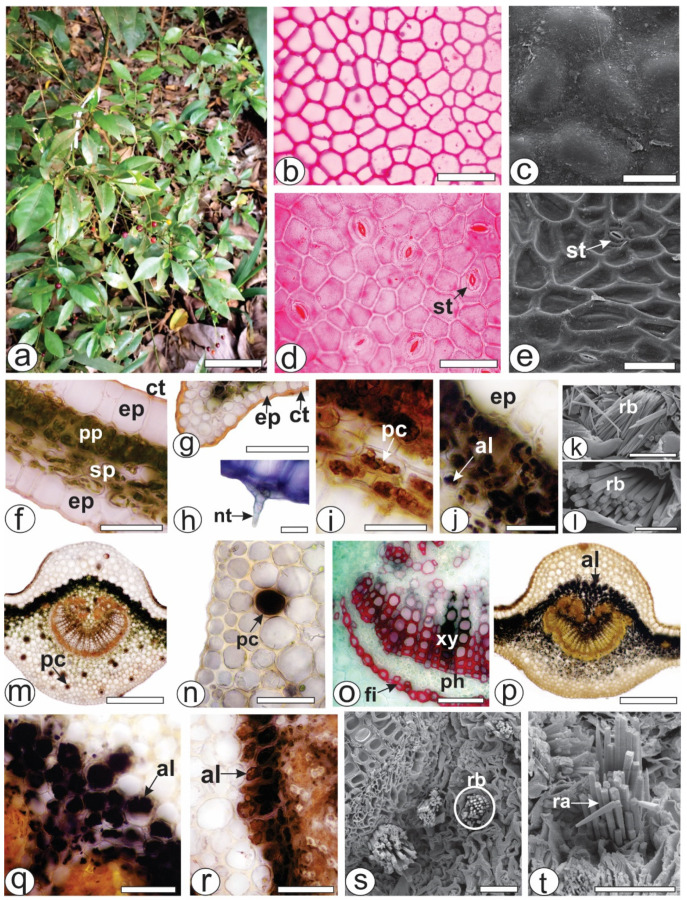
Morphoanatomy of *Psychotria capillacea* leaves. ((**a**) plant inhabits). ((**b**,**d**,**f**–**j**,**m**–**r**): light microscopy; (**c**,**e**,**k**,**l**,**s**,**t**): FESEM). (**b**–**e**) Leaf surface view. (**f**–**t**). Transverse sections. (**f**–**l**) Mesophyll. (**m**–**t**) Midrib. (al, alkaloids; ct, cuticle; ep, epidermis; fi, fibers; nt, nonglandular trichome; pc, phenolic compound; ph, phloem, pp, palisade parenchyma; ra, raphides; rb, raphides bundle; sp, spongy parenchyma; st, stomata; xy, xylem). Scale bar: (**a**) = 10 cm; (**m**,**o**) = 300 µm; (**b**,**d**–**g**,**n**,**p**–**r**) = 50 µm; (**c**,**i**–**k**,**s**,**t**) = 25 µm; (**h**,**l**) = 10 µm.

Both of the epidermal surfaces, on the *P. capillacea* (Figure 1a) leaves, had straight anticlinal walls (Figure 1b,d). The leaves were hypostomatic with paracytic stomata (Figure 1d,e). In the cross-section, the leaves were dorsiventral, showing one layer of palisade parenchyma and approximately four strata of spongy parenchyma (Figure 1f). The epidermis was unilayered and outwardly covered by a thin and smooth cuticle that reacted with Sudan Black and Sudan III (Figure 1f,g). Simple, pluricellular, nonglandular trichomes (Figure 1h) were rarely observed on the abaxial side. Phenolic compounds (Figure 1i) and alkaloids (Figure 1j) were detected in the chlorenchyma. The blue bundles (Figure 1k) consisted of four-sided crystals, squared in the cross-section (Figure 1l) and pointed at the ends (Figure 1k).

The midrib was biconvex (Figure 1m,p), and the vascular system was organized into open arcs with curved ends; on the inside of the bundle, the extremities were fragmented, forming small groups of xylem and phloem elements that were distributed in the innermost region (Figure 1m,p). A sheath of perivascular fibers that reacted positively to phloroglucinol/HCl (Figure 1o) surrounded the vascular bundle. Beneath the epidermis, annular or angular collenchyma was present on both sides (Figure 1m,p). The palisade parenchyma was continuous in the adaxial region (Figure 1m,p). Phenolic compounds were detected by potassium dichromate (Figure 1m) and ferric chloride (Figure 1n) solutions in the phloem and idioblasts dispersed in the ground parenchyma of the midrib (Figure 1m,n). Alkaloids that reacted with Dragendorff’s (Figure 1p,q) and Wagner (Figure 1r) reagents were detected inside the cell vacuoles. These genes were detected in the chlorenchyma cells (Figure 1p–r) and in the ground parenchyma of the midrib (Figure 1p). As previously described for the lamina, the Raphides bundles were found in the ground parenchyma (Figure 1s,t).

**Figure 2:**
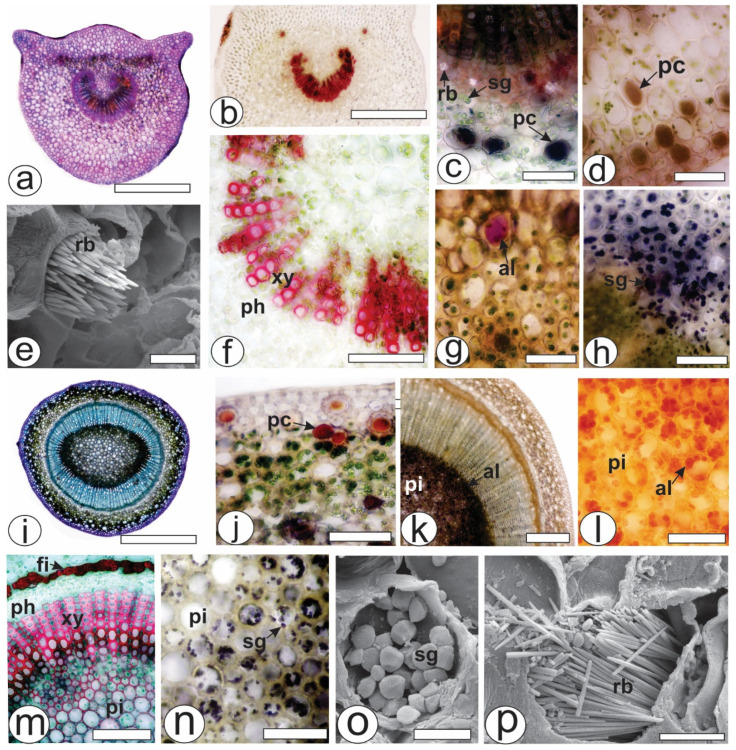
Anatomy of the leaves and stems of *Psychotria capillacea* in the cross-section ((**a**–**d**,**f**–**n**): light microscopy; (**e**,**o**,**p**): FESEM). (**a**–**h**) Petiole (**i**–**p**). Stem. (al, alkaloids; fi, fibers; pc, phenolic compound; ph, phloem, pi, pith; rb, raphide bundle; sg, starch grains; xy, xylem). Scale bar: (**a**,**i**) = 500 µm; (**b**) = 300 µm; (**k**) = 200 µm; **c**,(**d**,**f**–**h**,**j**,**l**–**n**) = 50 µm; **e**,**o**,**p** = 10 µm.

The petiole, which was in a cross-section in the middle of the structure, was biconvex with lateral projections (Figure 2a). The unilayered epidermis was covered by a thin cuticle. Angular collenchyma was observed beneath the epidermis. The vascular system was represented by a collateral bundle in the open arc with two accessory bundles (Figure 2b). The conductive elements of the xylem were in a radial series separated by parenchyma cells and reacted with phloroglucinol/HCl (Figure 2f). Raphides (Figure 2e), phenolic compounds (Figure 2c,d), alkaloids (Figure 2g) and starch grains (Figure 2h) were found in the ground parenchyma.

During transection, the stem was eye-shaped (Figure 2i), and the epidermis was uniseriate and covered by a thick cuticle. Phenolic compounds were found in the cortex (Figure 2j), whereas alkaloids were present in great amounts in the pith (Figure 2k,l). Fibers adjoined the external phloem (Figure 2m), and together with the xylem and pith, the cells reacted positively to the lignified elements (Figure 2m). The xylem was composed of vessel elements with helical thickenings and was arranged in radial rows separated by lignified parenchyma cells. Several starch grains were observed in the pith (Figure 2n,o). Raphide bundles were also found in the stem (Figure 2p).

**Figure 3:**
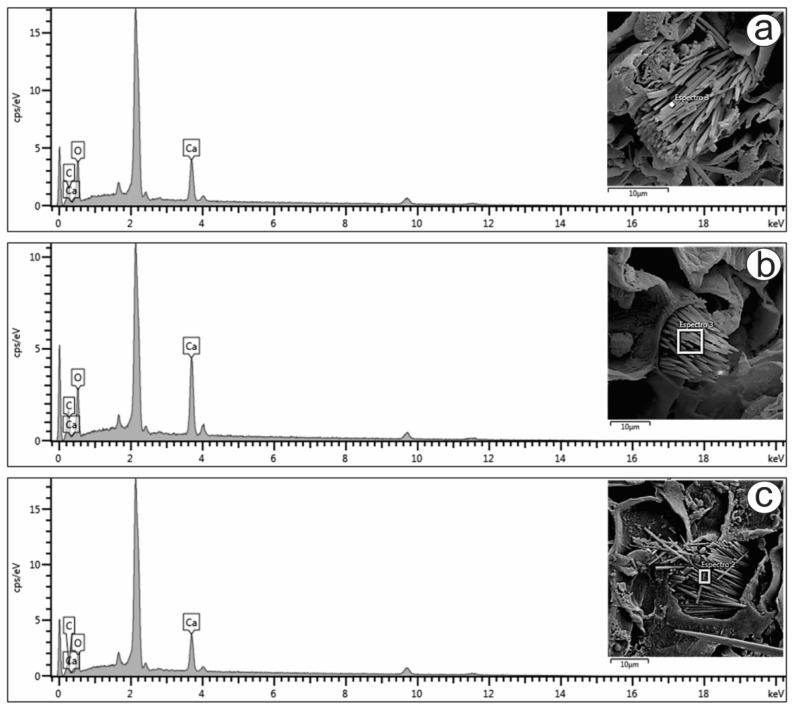
FESEM image and EDS spectrum of raphide crystals in the (**a**) mesophyll, (**b**) petiole and (**c**) stem of Psychotria capillacea.

Only raphides were found in the present study, and their elemental compositions were analyzed (Figure 3a–c). The energy-dispersive X-ray spectroscopy (EDS) spectra of the raphides localized in the mesophyll (Figure 3a), petiole (Figure 3b) and stem (Figure 3c) showed prominent peaks for carbon (16.5, 14.8 and 13.2%, respectively), oxygen (58.6, 52.5 and 49.8%, respectively) and calcium (24.8, 32.6 and 37.0%, respectively). The peaks varied slightly in the different spectra, as shown in Figure 3. The atomic proportions of Ca, C and O (1:2:4) obtained from the spectra indicated that the crystals were composed of calcium oxalate (CaC_2_O_4_), which is also suggested by the crystal shape.

### 2.2. Chemical Composition

#### Molecular Networking

**Figure 4:**
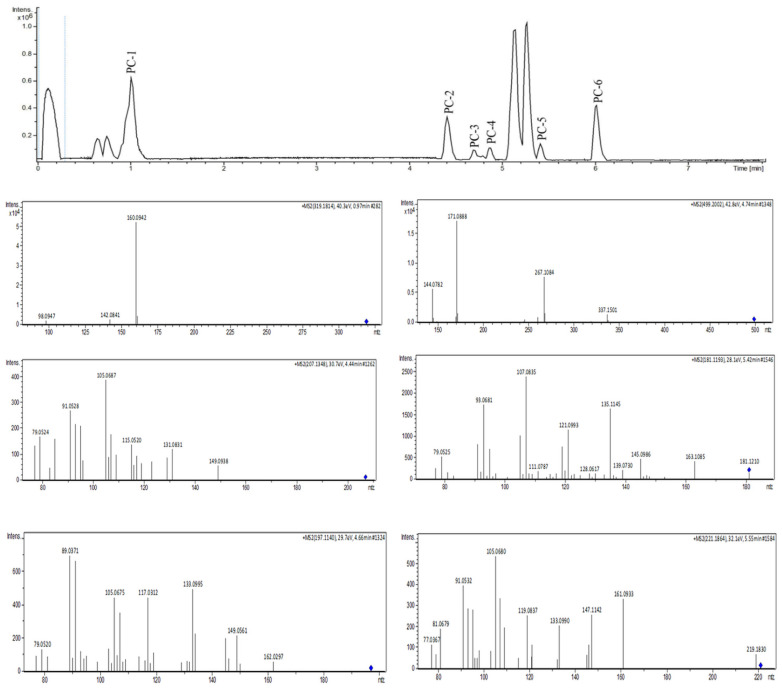
UHPLC-MS/MS analysis of MEPC in positive ionization mode.

**Figure 5:**
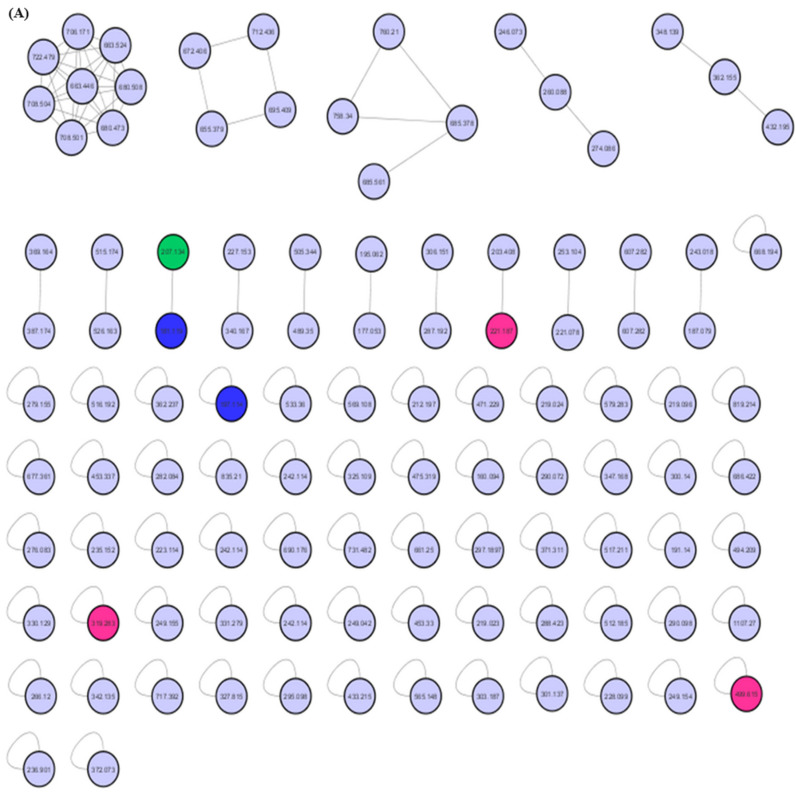

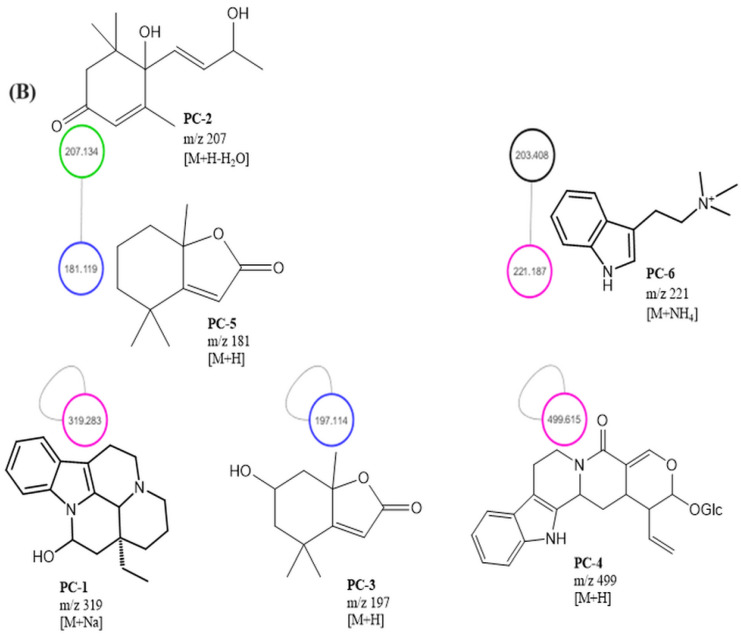
MEPC molecular networking via UHPLC–MS/MS analysis. (**A**) Molecular network. (**B**) Clusters with MS-Clusters with a parental mass tolerance of 2.0 Da and an MS/MS fragment ion tolerance of 0.5 Da.

The MEPC was analyzed by dereplication by ultraperformance liquid chromatography–high-resolution tandem mass spectrometry UHPLC-MS/MS in positive ion mode. Subsequently, the obtained fragmentation data were processed to generate a molecular network through the GNPS platform to identify the six compounds (Figure 4 and Figure 5). 

The molecular network for MEPC was generated from 106 parent ions and visualized as nodes (Figure 5A). Based on the cosine similarity, 16 clusters were formed. The clusters associated with alkaloids (pink nodes), terpenes (green node) and terpene lactones (blue nodes) are highlighted in Figure 5B. Molecular networking was used to cluster the detected aglycones according to their specific fragmentations.

Mass-spectrometry-based molecular networking was used as a dereplication strategy, leading to the putative identification of three known indole alkaloids, vincanol (PC-1), vincosamide (PC-4) and N,N,N-trimethyltryptamine (PC-6); one (megastigmane-type) sesquiterpene vomifoliol (PC-2); and two terpene lactones, loliolide (PC-3) and dihydroactinidiolide (PC-5) (Figure 4 and Figure 5). These compounds were identified based on the similarity between their fragmentation patterns and those of compounds in the literature (Table 1).

Dragendorff’s reagent was used to confirm that AF was alkaloid-positive, as orange-colored halos and purple- and blue-colored halos were observed after H_2_SO_4_/anisaldehyde/acetic acid solutions were applied with heating, indicating the presence of terpenes and sugars. The HPLC-DAD chromatogram obtained for AF showed the presence of the following main peaks: tr: 20.86 min (absorption bands at 260–280; 330–360 nm), tr: 23.08 min (280–290 nm), tr: 23.61 min (250–255; 270–290; 330–350 nm) and tr: 26.28 min (260–290; 340–350 nm) (Figure S1—Supplementary Material). The ^1^H nuclear magnetic resonance (NMR) spectrum revealed characteristic signs in the δ_H_ 7.0–8.0 ppm in AF (Figure S2—Supplementary Materials).

### 2.3. Anti-Inflammatory Activity

**Figure 6:**
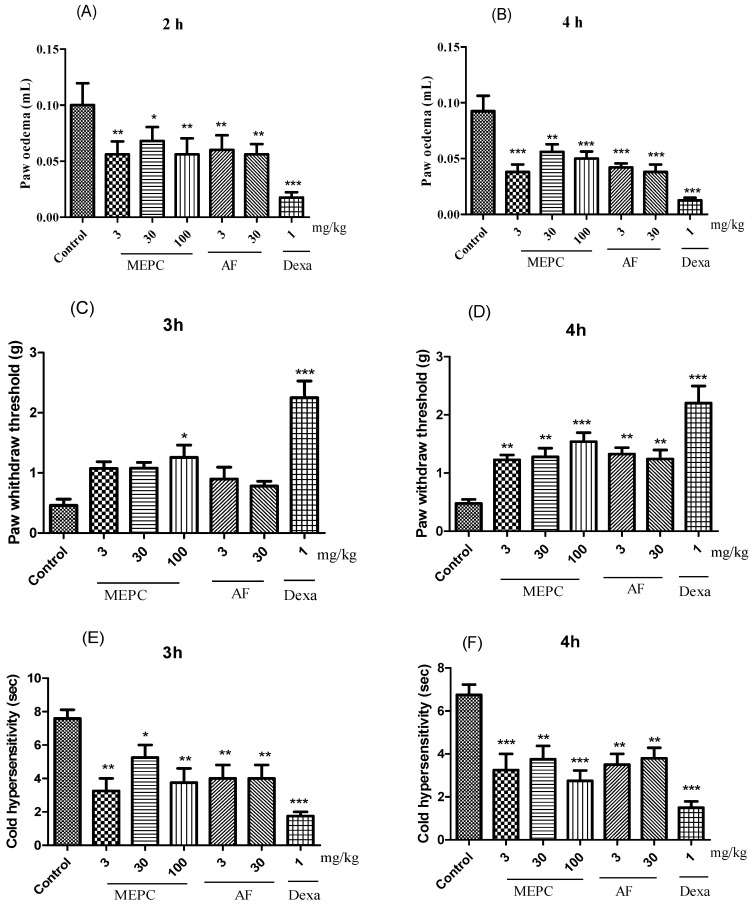
Effects of MEPC (3, 30 and 100 mg/kg) and AF (3 and 30 mg/kg) on paw edema (**A**,**B**), mechanical hyperalgesia (**C**,**D**) and cold thermal activity (**E**,**F**) after induction with carrageenan. The bars express the mean ± SEM of 7 animals, compared with the control vs. treated group. * *p* < 0.05; ** *p* < 0.01; *** *p* < 0.001; one-way ANOVA followed by the Student–Newman–Keuls test.

Three parameters, edema, mechanical hyperalgesia, and cold allodynia, were analyzed in response to the treatment of carrageenan-induced paw inflammation with the extract (MEPC; 3, 30 and 100 mg/kg) and the alkaloid fraction (AF; 3 and 30 mg/kg) obtained from *P. capillacea*. In the edema model, compared with the control treatment, MEPC (3, 30 and 100 mg/kg) and AF (3 and 30 mg/kg) significantly decreased edema formation. After 2 h, inhibition values of 44 ± 5%, 32 ± 4%, 44± 6%, 40± 5% and 43 ± 3%, respectively, were obtained; after 4 h, these values were 58 ± 4%, 39 ± 3%, 45 ± 3%, 54 ± 2% and 58 ± 3%, respectively (Figure 6A,B), confirming the antiedematogenic potential of these two materials. The positive control dexamethasone (Dexa, 1 mg/kg) significantly reduced edema at 2 h (82 ± 3%) and 4 h (86 ± 1%) (Figure 6A,B).

In the mechanical hyperalgesia test, treatment with MEPC (100 mg/kg) exhibited the greatest inhibitory effect in mice, in which hyperalgesia was inhibited by 64 ± 6% at 3 h and 70 ± 3% at 4 h (Figure 6C,D). Dexa (1 mg/kg) reduced the mechanical hyperalgesia induced by carrageenan by 80 ± 1% after 3 h (Figure 6C) and by 78 ± 3% after 4 h (Figure 6D). At 4 h, MEPC (3 and 30 mg/kg) and AF (3 and 30 mg/kg) also significantly reduced mechanical sensitivity by 60 ± 3%, 62 ± 2%, 63± 1% and 61± 2%, respectively (Figure 6D).

In the allodynia sensitivity assay, allodynia was observed for 30 s by evaluating paw licking, shaking, or rubbing behaviors (Figure 6E,F). MEPC (3 and 100 mg/kg) reduced the sensitivity to cold acetone stimulus. At 3 h, reductions of 57 ± 4% and 50 ± 5% were obtained, respectively, and at 4 h (Figure 6E) reductions of 61 ± 5% and 68 ± 4% were obtained, respectively (Figure 6F). AF (3 and 30 mg/kg) also significantly reduced the cold response to acetone in mice, as indicated by inhibition ≥ 47% at both 3 and 4 h (Figure 6E,F).

### 2.4. Learning and Memory Processes in Mice with Scopolamine (SCP)-Induced Dementia and AChE Inhibitory Activity

**Figure 7:**
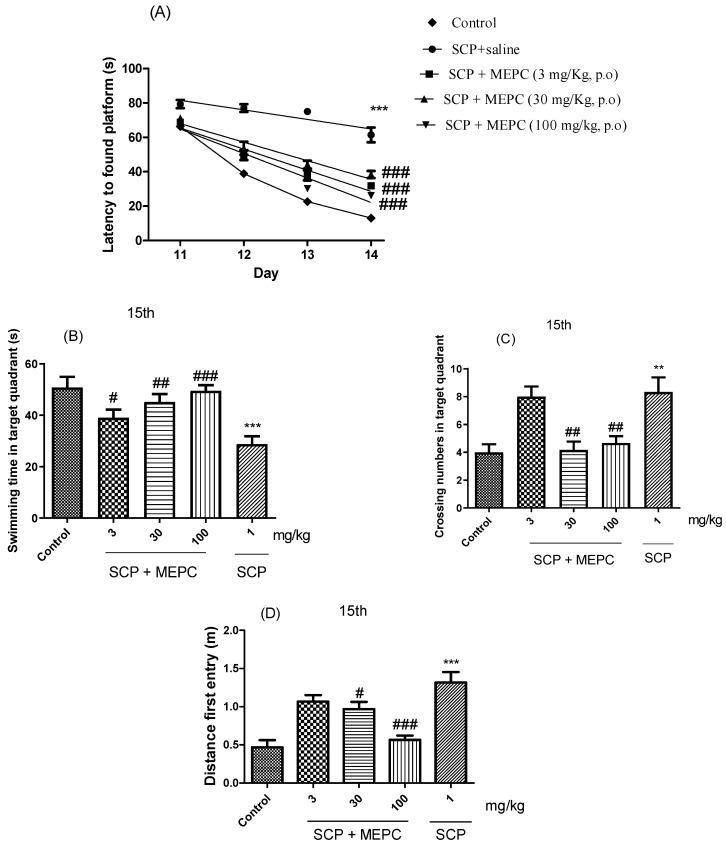
Effects of MEPC on SCP-induced cognitive deficits. Control (saline), scopolamine (SCP, 1 mg/kg + saline) or scopolamine plus extract (SCP + MEPC, 3 mg/kg), (SCP + MEPC, 30 mg/kg) or SCP + MEPC, 100 mg/kg) were used in the MWM test. (**A**) Escape latency in the 15th acquisition trial; (**B**) time in the target quadrant; (**C**) number of entries in the target quadrant in the 15th; (**D**) distance to first entry in the 15th acquisition trial. The data are presented as the mean ± SEM; *n* = 7. ** *p* < 0.01, *** *p* < 0.001 compared with the control group; # *p* < 0.05, ## *p* < 0.01, ### *p* < 0.001 compared with the SCP + saline group.

The spatial and learning memory of the mice were assessed using a water maze. As shown in Figure 7A, the escape latency in the acquisition trial over 4 days (the time from when the mouse was placed in the water to when the mouse found the platform was recorded as the escape latency) decreased throughout the acquisition test for all groups, indicating the ability of the mice to locate the platform. After acquisition training, all the groups were subjected to the probe trial phase, during which the platform was removed from the tank to test the spatial memory of the animals. Treatment with MEPC (3, 30 or 100 mg/kg) effectively reduced the escape latency on the fourth day by ≥38 s compared to that in the SCP treatment group (Figure 7A). The mean escape latency of the mice in the control group decreased from 66 s to 14 s during the four training days; however, the mean escape latency of the mice in the SCP + saline group decreased from only 79 s to 61 s over the same period.

The swimming time in the target quadrant, the number of crossings and the distance of the first entry on day 15 were used to analyze memory formation (Figure 7B–D). The time spent swimming in the target quadrant was markedly lower in the SCP group (1 mg/kg) than in the control (saline) group. Treatment with MEPC (3, 30 or 100 mg/kg) significantly increased the swimming time in the target quadrant compared to that in the SCP group (Figure 7B). A higher MEPC dose (100 mg/kg) had a significant effect on the serum ALB concentration, suggesting a dose-dependent response (Figure 7B). As shown in Figure 7C,D, compared with SCP, MEPC (100 mg/kg) significantly increased the number of entries into the target quadrant and had a significant effect on the precision of the first entry into the target quadrant; therefore, the formation of memories increased, and cognitive impairments were ameliorated.

**Figure 8:**
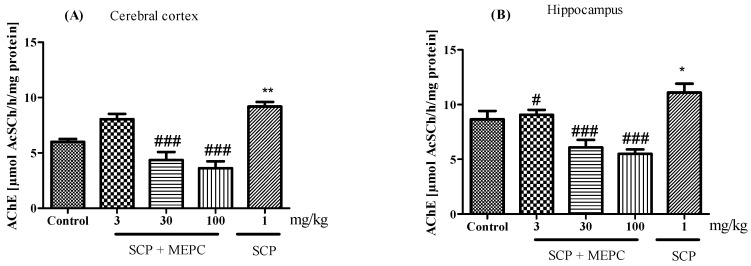
Effects of MEPC (3, 30 and 100 mg/kg) on AChE activity in the (**A**) cerebral cortex and (**B**) hippocampus of 15-day-old mice. The control (saline), scopolamine (SCP, 1 mg/kg + saline), SCP + MEPC (3 mg/kg), SCP + MEPC (30 mg/kg) and SCP + MEPC (100 mg/kg) groups were used. The data are presented as the mean ± SEM; *n* = 7. * *p* < 0.05, ** *p* < 0.01 compared with the control group; # *p* < 0.05, ### *p* < 0.001 compared with the SCP group.

AChE activity was altered in both brain structures evaluated (Figure 8A,B). AChE activity in the brain was markedly greater in the SCP group than in the control group (Figure 8). After treatment with MEPC (30 and 100 mg/kg), the AChE activity in the cerebral cortex was significantly lower (52 ± 3% and 60 ± 3%, respectively) than that in the SCP group, and the AChE activity in the hippocampus was 45 ± 3% and 50 ± 1%, respectively (Figure 8A,B). Interestingly, compared to the SCP group, MEPC (3 mg/kg) did not inhibit AChE activity.

## 3. Discussion

Considering the research interests of our laboratory and that *Psychotria* is used for pain- and inflammation-related symptoms in folk medicine, we first assessed the micromorphology and histochemistry of the leaves and stems of this plant in an attempt to support its use a medicinal herb. Then, before conducting inflammation and behavioral tests, we performed a dereplication study by UHPLC-MS/MS and molecular networking to support the biological tests.

Epidermal surfaces with straight anticlinal walls and leaf hyphae with paracytic stomata are characteristics frequently found in *Psychotria* species [22]. Some *Psychotria* species are glabrous [22], and others, such as *P. viridis* Ruiz & Pav., have stellate trichomes [23]. The presence and type of trichomes are important factors for identifying related species [24]. Crystals found in *P. capillacea* were reported for the *Psychotria* genus [25].

The midrib is biconvex, as described for various *Psychotria* species; however, a flat-convex shape was observed for *Psychotria carthagenensis* Jacq. [22]. The vascular system showed the same pattern in *P. carthagenensis*, *P. glaziovii* [22]) and *P. viridis* [23]. The palisade parenchyma was continuous in the adaxial region, as reported for other *Psychotria* species [22].

The petiole showed different outlines than those found in other *Psychotria* species [22]. The outline and vascular pattern of the petiole are important features in the differentiation of species within a specific genus, as reported for *Piper* [26] and *Baccharis* [27]. The vascular bundle of the petiole is surrounded by a sheath of perivascular fibers in some species of *Psychotria* [13], which was not found in the present study. The presence of raphides, phenolic compounds, and alkaloids in the ground parenchyma was also reported in *P. viridis* [23].

Druses, prismatic materials, styloids and raphides have been reported in *Psychotria* species [22,28]. The atomic proportions of Ca, C and O (1:2:4) obtained from the spectra indicated that the crystals were composed of calcium oxalate (CaC_2_O_4_), which was also suggested by the crystal shape. The presence or absence of crystals and their type and chemical composition support plant taxonomy. Crystals have been described in some studies as calcium oxalate by using EDS [29,30,31,32,33].

Three known indole alkaloids were putatively identified through the application of mass-spectrometry-based molecular networking as a dereplication strategy in the MEPC (Figure 4 and Figure 5). These alkaloids may be present in AF, based on DAD analysis and NMR data (Figures S1 and S2—Supplementary Materials). The presence of bands in the region of 220–270, 270–300, and 310–360 (DAD) is related to the β-carboline chromophore, indicating the presence of indole β-carboline alkaloids in this fraction and in 1H NMR, which showed characteristic signs of the indole nucleus in the δ_H_ 7.0–8.0 ppm and also unshielded hydrogens in the region of 7.90 to 8.00 ppm, which could be related to H-4 of the β-carboline ring when this nucleus is substituted in position-3 (Figures S1 and S2—Supplementary Material). These data can confirm that β-carboline alkaloids (PC-1 and PC-4) are present in the AF.

These results correspond with the micromorphology and histochemical findings of the present study. Chemical studies carried out by our group have revealed the presence of indole alkaloids, such as vincosamide in *P. leiocarpa* [11], tryptamine-related alkaloids in *P. brachybotrya* [34] and vomifoliol and loliolide in *Palicourea tomentosa* (synonym *Psychotria poeppigiana*) [35].

A recent study in the literature reported that some of the compounds isolated from *P. capillacea* showed anti-inflammatory and neuroprotective properties. Dihydroactinidiolide (a monoterpene lactone, a structural analogue of loliolide) has neuroprotective effects under escalated oxidative stress conditions in scopolamine-induced amnesic mice via oxidative stress quenching, enhancing antioxidative enzyme activity, enhancing BDNF and synaptophysin mRNA levels, and reducing the expression of the apoptotic protein Bax [36]. Loliolide abates LPS-induced inflammation via the TLR-mediated NF-κB and MAPK pathways in macrophages [37]. Vincosamide reduces chemically induced inflammation in mice and inhibits AChE activity [11]. Vomifoliol has shown antioxidant, anti-inflammatory and AChE inhibitory activities [38].

In this study, three parameters, edema, mechanical hyperalgesia, and cold allodynia, were analyzed with respect to the extract (MEPC) and fraction (AF) obtained from *P. capillacea* after carrageenan was used to induce paw inflammation. Edema, fever, redness and pain are the most common signs that characterize acute inflammation and are widely described; in addition, these signs are used to analyze the anti-inflammatory agents involved in the inflammatory process [11,39]. In this study, the extract and fraction negatively modulated carrageenan-induced spontaneous pain in the paw and mechanical and cold hyperalgesia models.

Many rodent models, such as von Frey hairs and sensitivity to brushes for mechanical allodynia, pin pricking for mechanical hyperalgesia, and hot plate and radiant heat for heat pain, have been developed and characterized using standardized limb withdrawal measures as surrogates for the sensory threshold or responsiveness to innocuous or noxious stimuli [11,40,41,42]. Two models were used in this study. According to the mechanical hyperalgesia test, MEPC and AF inhibited mechanical sensitivity. According to the sensitivity allodynia assay, MEPC (3 and 100 mg/kg) potentially reduced the sensitivity to the cold stimulus acetone.

Preparations obtained from *Psychotria* were assayed in vitro and in clinical models. In folk medicine, these plants are used for inflammation-related symptoms. However, phytochemical and biological information from only two groups of *P. capillacea* has been reported in the literature [3,4]. *Palicourea tomentosa*, *Psychotria leiocarpa*, *P. nuda*, *P. nemorosa*, *P. brachypoda*, *P. colorata* and *P. myriantha* have been reported to have anti-inflammatory and analgesic effects and impair learning and memory acquisition [11,12,35,43,44,45]. However, only two studies of *P. capillacea* in the literature have reported phytochemical and biological information [3,4].

Several studies have reported that indole alkaloids isolated from *Psychotria* act on the central nervous system [13,14,15,46,47,48,49]. For the first time, we evaluated the effects of MEPC for fourteen days using the Morris water maze (MWM) test to assess the spatial and learning memory of the mice in a water maze. In this model, cognitive impairment was induced by SCP, and SCP-induced cognitive deficits were reversed by oral treatment with three doses of MEPC.

The swimming time in the target quadrant, the number of crossings and the distance of the first entry on day 15 were used to analyze the formation of memory. The reduction in swimming time in the target quadrant and the number of entries in the target quadrant may indicate cognitive impairment in the SCP group. Treatment with MEPC seemed to prevent cognitive impairment, as memory formation increased and cognitive impairments were ameliorated. These effects may result from the flavonoids and alkaloid compounds in MEPC due to their polarity.

Next, we evaluated the inhibitory effects of MEPC on AChE activity in brain tissue, the cerebral cortex and the hippocampus, which are involved in learning, the formation of memory, and the storage of memory. Treatment with MEPC reduced AChE activity in the brain, indicating its neuroprotective effect.

Thus, the use of medicinal plants is an important strategy for providing primary health care, and in line with the World Health Organization and the United Nations Children’s Fund (UNICEF), the Unified Health System has developed public policies and national regulations regarding the use of traditional remedies with proven efficacy and the possible incorporation of this knowledge into primary health care activities. Dementia comprises a set of symptoms including pronounced memory loss, and Alzheimer’s disease is one of the most common causes of dementia, which is one of the main reasons for human disability throughout life. Increasing life expectancy and ageing populations are additional reasons for identifying additional therapeutic options. Thus, investigating the pharmacological activities of natural products obtained from medicinal plants is essential in the search for novel neuroprotective agents since most neurological disorders focus on symptomatic relief only. This fact demonstrates the originality of and the need for this study.

## 4. Materials and Methods

### 4.1. Chemicals

Carrageenan, dexamethasone, scopolamine hydrobromide, and donepezil hydrochloride monohydrate were purchased from Sigma—Aldrich Co. (St. Louis, MO, USA). All chemical reagents and solvents used were of analytical grade (Vetec, Rio de Janeiro, Brazil).

### 4.2. Plant Material

Leaves and stems of *P. capillacea* were collected in March 2019 in the region of Dourados (S 22°17′38.4″, W 54°95′94.2″), Mato Grosso do Sul, Brazil. The strain was identified by one of the authors (Z.V. Pereira/UFGD) and deposited in the herbarium of this institution (5008); this research was registered at SisGen A51F665.

### 4.3. Microscopy Procedure

The methods employed for the light microscopy (LM), field emission scanning electron microscopy (FESEM) and energy-dispersive X-ray spectroscopy (EDS) analyses of the leaves and stems of *P. capillacea* are described in detail in a previous paper by Brito et al. [50].

### 4.4. Histochemical Analyses

Freehand cross sections were created with fresh material and exposed to phloroglucinol/HCl to detect traces of lignin and iodine–iodide solution (2%) to identify starch grains [51]; Wagner’s [52] and Dragendorff’s [53] reagents to identify alkaloids; potassium dichromate (10%) and ferric chloride (1%) to detect phenolic substances [54]; Sudan III and Sudan black B to detect lipophilic compounds [55]. Appropriate controls were examined in these tests. Photomicrographs were captured using an Olympus CX 31 light microscope attached to a C-7070 control unit. The microscopic data were analyzed at the Laboratory of Pharmacognosy at the State University of Ponta Grossa (UEPG) to obtain a comprehensive description of the leaf and stem tissues.

### 4.5. Chemical Analyses

#### 4.5.1. Extraction and Fractionation of the Methanolic Extract of *P. capillacea*

The air-dried leaves and stems of *P. capillacea* (432 g) were extracted by maceration with methanol (MeOH, 8 × 4 L) at room temperature, followed by filtration, evaporation of the solvent under vacuum and lyophilization to obtain the methanolic extract (MEPC) (47.2 g). Part of this extract (30 g) was fractionated using acid—base extraction by treatment with HCl (5%) solution and extracted with dichloromethane (CH_2_Cl_2_). The acidic aqueous phase was basified with NH_4_OH to pH 9–10 and extracted with CH_2_Cl_2_ and CH_2_Cl_2_: MeOH (2:1). The organic fractions were combined, washed with water, dried over calcium chloride (CaCl_2_), and filtered. Then, the solvent was evaporated to furnish the alkaloid fraction (AF), which was subsequently analyzed by TLC plates (silica gel 60 G or 60 GF_254_, Merck) eluted with a mixture of chloroform and 30% methanol and visualized by UV irradiation at 254 and 366 nm, and/or by spraying with H_2_SO_4_/MeOH (1:1), H_2_SO_4_/anisaldehyde/acetic acid (1:0:50 mL), or Dragendorff’s solution, followed by heating at 100 °C. MEPC and AF were stored at 4 °C until testing and analysis.

#### 4.5.2. Chemical Analysis of MEPC by UHPLC—MS/MS, Molecular Networking and Alkaloidal Fraction (AF)

The analyses were carried out on a UHPLC system (Shimadzu, Nexera X2, Kyoto, Japan) coupled to a quadrupole time-of-flight (QTOF) mass spectrometer (Bruker, model Impact II, Bruker Daltonics Corporation, Billerica, MA, USA). An electrospray ionization (ESI) source was used in positive mode and scan operation mode, scanning from *m*/*z* 50 to 1300. The data-processing software used was Data Analysis 4.3 (Bruker). Chromatographic separations were performed using a UHPLC system equipped with a reversed-phase column (Symmetry C18) maintained at a temperature of 40 °C. Water (solvent A) and 0.1% formic acid in acetonitrile (solvent B) were used as the mobile phases. The flow rate used in the UHPLC—MS/MS analyses was 0.2 mL/min, and the sample injection volume was 3 µL. Analyses of the generated data and subsequent characterization of the known compounds present in MEPC were based on the fragmentation profiles of each class of metabolites.

The UHPLC—MS/MS data were converted to mzXML format using Bruker’s data software (version 4.3; Bruker Daltonics, Billerica, MA, USA), converted into the Global Natural Products Social Networking (GNPS) platform [56] and submitted for molecular network formation. The data were clustered with MS-Clusters with a parental mass tolerance of 2.0 Da and an MS/MS fragment ion tolerance of 0.5 Da. The edge parameters were a cosine score greater than 0.7 and more than 2 matching peaks. The data were filtered by removing all MS/MS peaks within ±17 Da of the *m*/*z* precursor. The network was evaluated against the GNPS spectral libraries, and all corresponding sequences had a cosine score above 0.7 and at least 6 corresponding peaks. The network analysis products were exported from GNPS and analyzed via Cytoscape 3.10.2.

The AF was analyzed in an analytical LC system with a diode array detector (DAD) monitored at λ = 190–500 nm. The LC column was a C-18 (25 cm × 4.6 mm; particle size, 5 μm; Luna, Phenomenex, Torrance, CA, USA). The flow rate and the injection volume were set as 1.0 mL/min and 10 μL. All chromatographic analyses were performed at 23 °C. Elution was carried out using phase methanol (A): water (B). The following gradient was applied: 5–100% (A) (3 min) and 100% B (10 min). At the end of the run, a return gradient of 100–5% for 5 min was used, with a waiting time of 15 min for column reconditioning. ^1^H NMR spectra were recorded on a Varian Mercury Plus BB spectrometer at 300 MHz using deuterated chloroform (CDCl_3_) as solvent, and tetramethylsilane (TMS) as internal standard.

### 4.6. Animals

The animal experiments were performed using male Swiss mice (25–35 g) provided by the Federal University of Grande Dourados (UFGD) biotherium. The animals were maintained on a 12 h light–dark cycle (lights on at 6:00 am) with 60–80% humidity and given free access to food (Nuvilab Cr-1) and water.

MEPC and AF was dissolved in saline solution (0.9% saline) for in vivo assays. The control group received only the vehicle (in 0.9% saline). All the above-described oral pretreatment regimens (or administrations) were performed via gavage.

### 4.7. Anti-Inflammatory Activity

Male Swiss mice (*n* = 7 animals/group) were treated orally with MEPC (3, 30 or 100 mg/kg), the AF (3 or 30 mg/kg) or vehicle (control group). Dexa (1 mg/kg) was administered subcutaneously and used as a positive control. All the groups received intraplantar injections of 50 µL of carrageenan (300 µg/paw) suspended in 0.9% sterile saline. The contralateral paw received 50 µL of saline and was used as a control.

Edema: Paw edema thickness was measured 2 and 4 h after carrageenan injection with a paw plethysmometer (PANLAB Harvard). The difference in paw volume measurements was quantified as edema [11].

Mechanical hyperalgesia: The mechanical sensitivity (g) of the hind paw was measured by determining withdrawal thresholds using an electronic version of the Von Frey test (Insight^®^, EFF 301, digital analgesiometer). Constant pressure was applied to the plantar surface of the right hind paw with an analgesiometer until the mice vocalized or the paw was removed; this measurement indicated the level of mechanical sensitivity induced by sensitization 3 and 4 h after carrageenan administration [11].

Cold thermal activity: The cold response was evaluated by the acetone test [11]. After the animals were housed on a suspended platform, twenty microliters of acetone was distributed on the plantar surface skin of their right hind paw. The duration of the test was 30 s, and the indicators were paw licking, shaking, or rubbing.

### 4.8. Neuroprotective Effects

#### Spatial Memory, Learning and AChE Inhibitory Activity

Male Swiss mice (*n* = 7 animals/group) were treated orally with MEPC (3, 30 or 100 mg/kg), and control animals were treated with the same volume of saline for 15 days. The SCP group was treated with SCP (1 mg/kg). On the 11th day, 60 min after extract administration, SCP was injected intraperitoneally into the mice for 4 consecutive days.

The Morris water maze (MWM) test equipment consisted of a circular pool (90 cm in diameter and 30 cm in height) filled with water (22 °C) to a depth of 14 cm. A platform (10 cm × 10 cm) was formed with acrylic and hidden 2 cm below the water surface at a fixed location. In this test, dye was added to make the background invisible so that the animals could not see the platform [57]. The MWM test was performed beginning on the 14th day of the experiment, 30 min after SCP injection. The animals underwent training/trials in the maze for 4 consecutive days; on the 15th day, the platform was removed, and a test was performed to assess related memory. Animal behaviors were monitored to examine the retention of spatial reference memory, considering the number of platform crossings and swimming time in the target quadrant in which the platform was previously placed.

At the end of the MWM test, the animals were anesthetized with a combination of anesthetics (xylazine/ketamine) and euthanized. Immediately after euthanasia, brain samples (cortex and hippocampus) were carefully removed from the skull and washed twice with ice-cold saline. Brain tissue from the cortex and hippocampus was homogenized in a solution of 10 mM Tris-HCl (pH 7.4), and the protein concentration in the homogenized samples was determined via Coomassie blue and measurement at 595 nm using bovine serum albumin as a standard. The protein concentration was adjusted for the cerebral cortex (0.6 mg/mL) and hippocampus (0.8 mg/mL) [58]. The AChE inhibitory activity of the brain homogenate was evaluated using the Ellman assay with minor modifications [59]. The reaction data were quantified on a microplate, and the absorbance was measured 8 times every 13 s in an ELISA spectrophotometer at 412 nm. The assay was performed in triplicate. The drug donepezil (0.1 mM) was used as a positive control. Enzyme activity was expressed as μmol ACSCh/h/mg protein.

### 4.9. Statistical Analyses

The data are presented as the mean ± standard error (SEM). Significant differences were determined via analysis of variance (ANOVA) followed by the Newman–Keuls test (GraphPad Prism Software 5.0). Differences were considered significant at *p* < 0.05.

## 5. Conclusions

This is the first in vivo biological study performed with MEPC, as well as the anatomy of the leaves and stems. In MEPC, it was possible to identify three indole alkaloids, one sesquiterpene (megastigmane-type), and two terpene lactones. The results revealed reduced inflammatory parameters in a carrageenan model (mechanical, cold sensitivity and edema formation); attenuated escape latency; increased number of crossings; an increase in swimming time in the target quadrant in mice with SCP-induced cognitive impairment; and inhibited AChE activity in the frontal cortex and hippocampus. Therefore, *P. capillacea* may exhibit potential neuroprotective, anti-inflammatory, and antihyperalgesic effects.

A comprehensive description of the leaf and stem anatomy and histochemistry of *P. capillacea* is presented. All the anatomical characteristics of *P. capillacea* should be evaluated to characterize this species. Histochemical tests confirmed the presence of lipophilic material, alkaloids, phenolic compounds, starch grains, and lignified elements. Further studies involving anatomical and phylogenetic analyses of other species are essential to better understand the evolution and taxonomy of *Psychotria*.

## Figures and Tables

**Table 1:** The main compounds observed from *P. capillacea* aerial parts (MEPC) by UHPLC—MS/MS in positive mode.

Compounds	tr/ min	Molecular Formula	*m/z*	Ion Precursor *m/z*	Error/ ppm	MS/MS
vincanol (PC-1)	0.97	C_19_H_24_N_2_O [M + Na]^+^	319.1786	319.1814	8.77	160; 142; 98
vomifoliol (PC-2)	4.44	C_13_H_20_NaO_3_ [M + H-H_2_0]^+^	207.1384	207.1348	7.37	149; 131; 115; 105; 91; 79
loliolide (PC-3)	4.66	C_11_H_17_O_3_ [M + H]^+^	197.1172	197.1140	6.23	162; 149; 133; 117; 105; 89
vincosamide (PC-4)	4.74	C_26_H_30_N_2_O_8_ [M + H]^+^	499.2075	499.2002	4.62	337; 267; 171; 144
dihydroactinidiolide (PC-5)	5.42	C_11_H_17_O_2_ [M + H]^+^	181.1223	181.1193	6.56	145; 135; 121; 93
N,N,N-trimethyltryptamine (PC-6)	5.55	C_13_H_19_N_2_ [M + NH4]^+^	221.1887	221.1864	7.20	161; 147; 133; 119; 105; 91; 81

## Data Availability

Data are contained within the article.

## Supplementary Materials

The following supporting information can be downloaded at: https://www.mdpi.com/article/10.3390/ph17050564/s1, Figure S1: The analyses by TLC plates (A) and (B) LC-DAD (UV/DAD; λ = 245 nm) from *P. capillacea*. Figure S2. ^1^H NMR spectrum (CDCl_3_-d_6_, 300 MHz) for the alkaloidal fraction (AF) obtained from *P. capillacea*.
