# Supplementary material for: A Comprehensive Description of the Anatomy and Histochemistry of Psychotria capillacea (Müll. Arg.) Standl. and an Investigation into Its Anti-Inflammatory Effects in Mice and Role in Scopolamine-Induced Memory Impairment
Source: Pharmaceuticals (Basel). 2024 Apr 28;17(5):564. doi: 10.3390/ph17050564 (PMC11123824; doi:10.3390/ph17050564)
Supplement: Supplementary file 1 [file pharmaceuticals-17-00564-s001.zip › pharmaceuticals-2901673-supplementary.pdf]

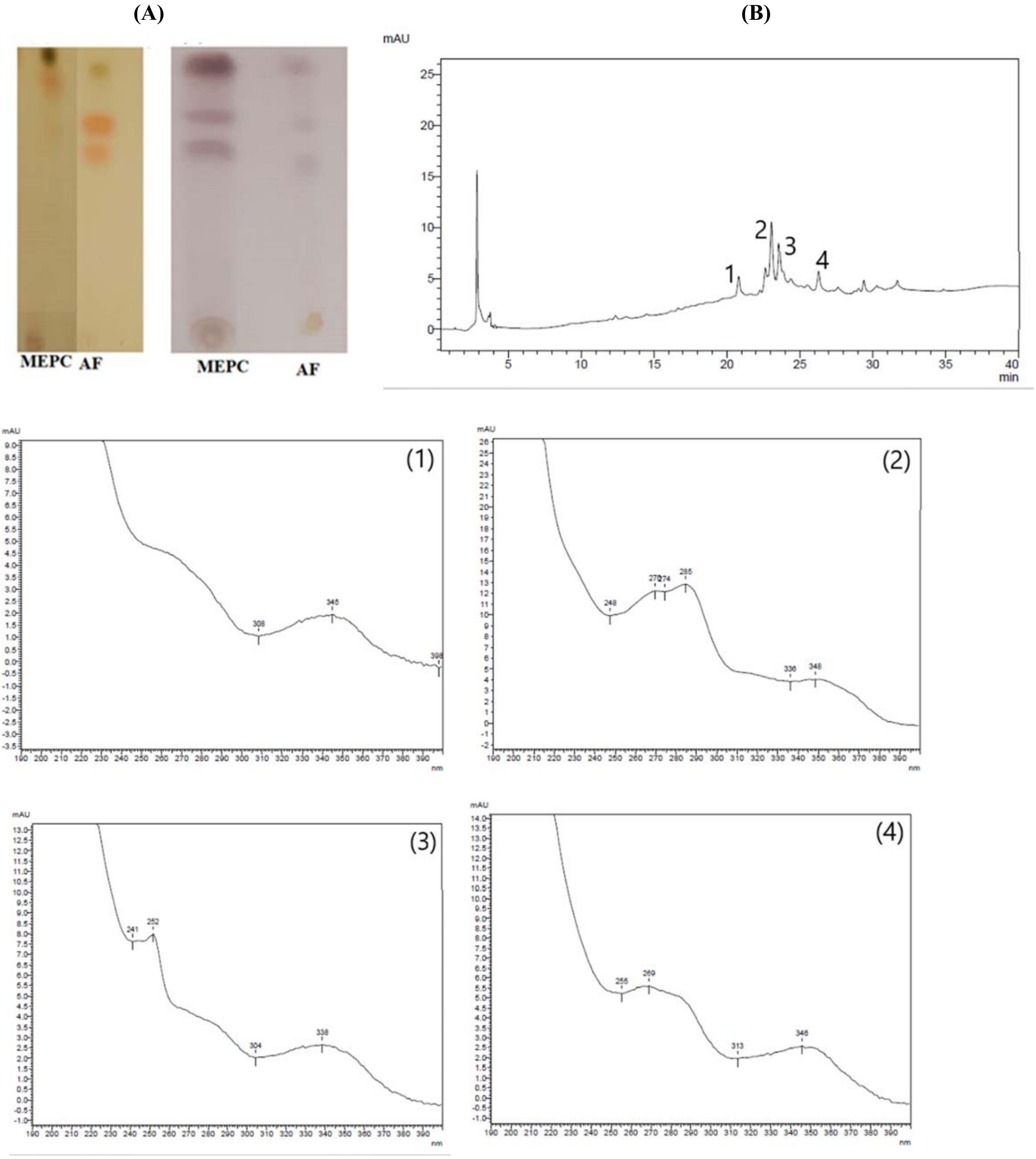

Figure S1. The analyses by TLC plates (**A**) and (**B**) LC-DAD (UV/DAD; λ = 245 nm) from *P. capillacea*.

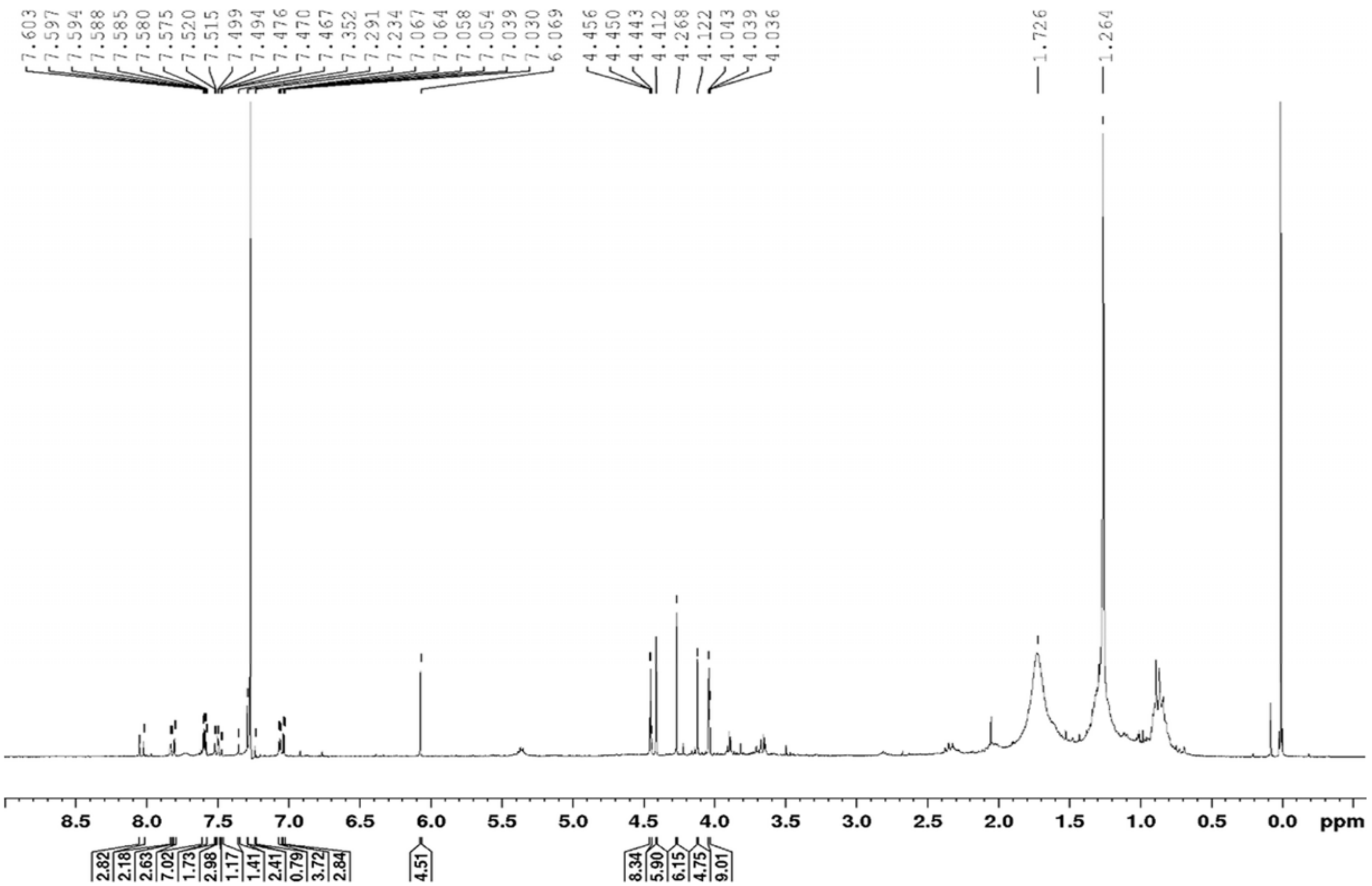
7.603
7.597
7.594
7.588
7.585
7.580
7.575
7.520
7.515
7.499
7.494
7.476
7.470
7.467
7.352
7.291
7.234
7.067
7.064
7.058
7.054
7.039
7.030
6.069
4.456
4.450
4.443
4.412
4.268
4.122
4.043
4.039
4.036
1.726
1.264
8.5
8.0
7.5
7.0
6.5
6.0
5.5
5.0
4.5
4.0
3.5
3.0
2.5
2.0
1.5
1.0
0.5
0.0
ppm
2.82
2.18
2.63
7.02
1.73
2.98
1.17
1.41
2.41
0.79
3.72
2.84
4.51
8.34
5.90
6.15
4.75
9.01

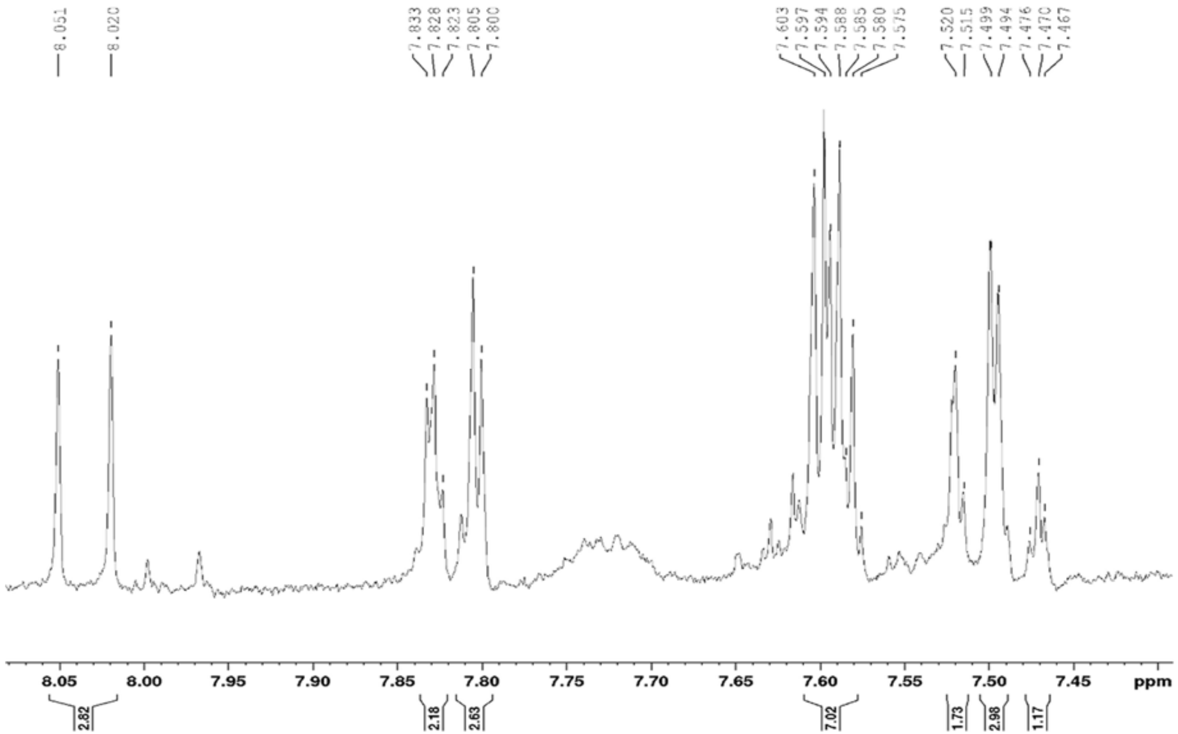

Figure S2. ^1H NMR spectrum ($CDCl_3$-d_6, 300 MHz) for the alkaloidal fraction (AF) obtained from *P. capillacea*.
